# Signatures of Selection in Fusion Transcripts Resulting From Chromosomal Translocations in Human Cancer

**DOI:** 10.1371/journal.pone.0004805

**Published:** 2009-03-12

**Authors:** Iñigo Ortiz de Mendíbil, José L. Vizmanos, Francisco J. Novo

**Affiliations:** Department of Genetics, University of Navarra, Pamplona, Spain; National Institute on Aging (NIA), National Institutes of Health (NIH), United States of America

## Abstract

**Background:**

The recurrence and non-random distribution of translocation breakpoints in human tumors are usually attributed to local sequence features present in the vicinity of the breakpoints. However, it has also been suggested that functional constraints might contribute to delimit the position of translocation breakpoints within the genes involved, but a quantitative analysis of such contribution has been lacking.

**Methodology:**

We have analyzed two well-known signatures of functional selection, such as reading-frame compatibility and non-random combinations of protein domains, on an extensive dataset of fusion proteins resulting from chromosomal translocations in cancer.

**Conclusions:**

Our data provide strong experimental support for the concept that the position of translocation breakpoints in the genome of cancer cells is determined, to a large extent, by the need to combine certain protein domains and to keep an intact reading frame in fusion transcripts. Additionally, the information that we have assembled affords a global view of the oncogenic mechanisms and domain architectures that are used by fusion proteins. This can be used to assess the functional impact of novel chromosomal translocations and to predict the position of breakpoints in the genes involved.

## Introduction

Most cancer cells display some type of chromosomal rearrangement. Whereas solid tumors usually display complex karyotypes with many different types of chromosomal rearrangements, many hematological malignancies and certain sarcomas display only one or a few aberrations, usually balanced chromosomal translocations, which in some cases have been shown to be the initiating event in tumor development [1], [2]. For this reason, chromosomal translocations are technically easier to characterize in hematological cancers. Extensive analysis of chromosomal translocations in human malignancies over the past three decades has revealed two main outcomes by which such rearrangements drive cancer progression: i) promoter exchange (mainly in lymphoid neoplasms), and ii) creation of chimeric genes that are translated as fusion proteins (myeloid leukemias and some solid tumors) [3]. Likewise, the consensus derived from these studies suggests that chromosomal translocations are the result of misrepaired DNA double-strand breaks (DSB) in somatic cells [4]–[7]. Chromosomal translocations resulting in chimeric fusion transcripts constitute an important group of reciprocal translocations that accounts for 20% of cancer morbidity in humans [3], and have the potential to initiate tumor growth because their protein products contain domains from both fusion partners. The presence of heterologous protein domains in the same chimeric protein results in deregulated biological activities that ultimately lead to cancer development.

Some of the balanced chromosomal translocations found in tumors are recurrent, in the sense that they are present in different patients with the same tumor type, or even in different tumor types [8]. Furthermore, characterization of fusion sequences at the molecular level in different patient samples has shown that, at least for a few genes, breakpoints tend to cluster in specific regions. As a result, the distribution of translocation breakpoints found in tumor samples follows a non-random pattern, with a few sites in which breakpoints are more frequent than expected by chance. Although several studies have addressed the potential role of nucleotide motifs and local sequence features as the cause for such recurrence [9]–[14], the importance of functional factors in delimiting the position of translocation breakpoints has not been tested experimentally. In this regard, a global analysis of chimeric fusion transcripts could show whether breakpoint recurrence might be the result of cellular selection for the functions encoded by specific domains that are present in the respective fusion proteins. Furthermore, the requirement to keep an intact reading frame in the fusion product could also contribute to explain the non-random distribution of translocation breakpoints across those genes.

In order to test this hypothesis, we have analyzed a comprehensive set of chromosomal translocations that create oncogenic fusion proteins in human malignancies, looking for signatures of functional selection. We compiled a catalogue of the protein domains encoded by those fusion proteins and visualized them as a network of interacting nodes, obtaining a global view of the protein domains that are brought together to the same fusion proteins. We also analyzed the reading frame of the fusion transcripts, in order to confirm that the original reading frames of the partner genes were kept in-frame in fusion transcripts in a proportion higher than expected by chance.

## Materials and Methods

Fusion sequences were obtained from TICdb version 2.1 (October 2007). TICdb is a freely available database of gene-mapped translocation breakpoints in cancer, which describes the genomic location of 1,445 translocation breakpoints, corresponding to 310 different genes, in hematological, mesenchymal and epithelial malignancies. The database was created using information from the *Mitelman Database of Chromosome Aberrations in Cancer* (available at the Cancer Genome Anatomy Project), two published catalogs of genes rearranged in cancer and our own searches [15]. Junction sequences of reciprocal translocations were mapped onto the reference sequence of the human genome using BLAST. All translocation breakpoints are thus referred to precise nucleotide positions or gene fragments (introns or exons) within specific *Ensembl* transcripts (Ensembl 38.36).

**Figure 1:**
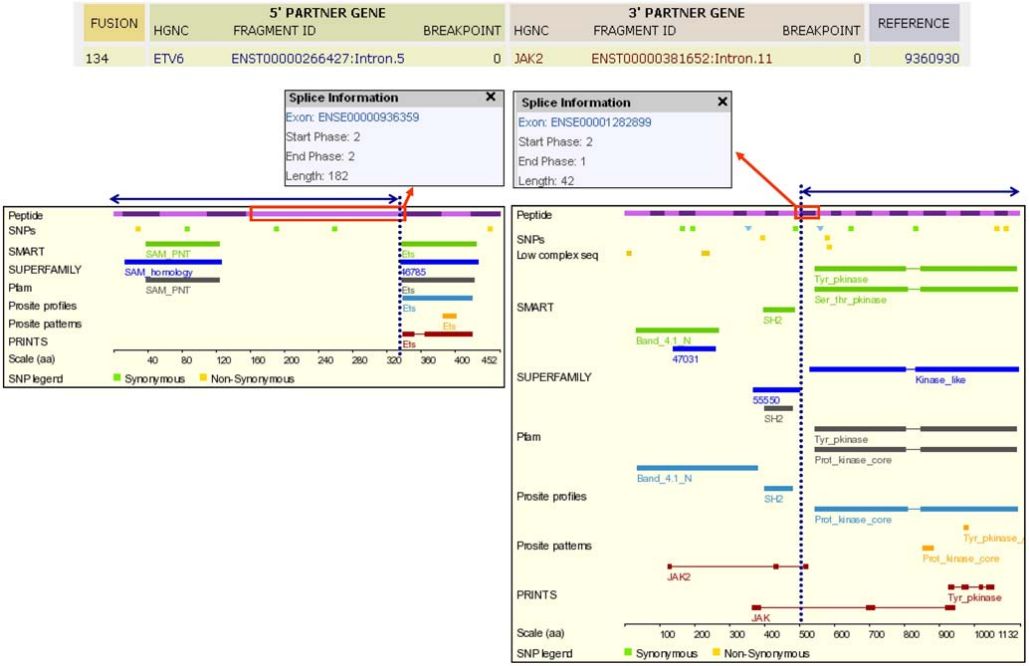
Procedure for the extraction of the data used in this study. For each fusion in TICdb (top rectangle shows part of the screenshot of a search for translocations involving ETV6) we went to the “Protein view” page of the respective transcripts (ENST00000266427 and ENST00000381652 in this example). The bottom left box shows the ETV6 protein with the aminoacids coded by each exon (blocks of alternating color), the position of protein domains annotated in several databases (SMART, SUPERFAMILY, PFAM, PROSITE and PRINTS), the location of the breakpoint (vertical dotted line) and the part of the peptide that is contributed to the fusion protein (horizontal line with double arrowhead). The same is shown for JAK2 in the bottom right box. In both cases, the exon flanking the fusion is highlighted (red rectangle), with its starting and ending reading frames shown in a box (“Splice information”).

The procedure followed is summarized in Figure 1. From TICdb, we obtained information about 699 different oncogenic gene fusions, excluding from further analysis all those translocations in which the oncogenic mechanism has been shown to be gene de-regulation by promoter exchange instead of the creation of a fusion protein. Likewise, 116 fusions in which at least one of the partner genes did not contribute a recognizable protein domain to the fusion protein were uninformative for the analysis of protein domain co-occurrences, and were thus excluded from the dataset because they are not eligible for the study. In total, we analyzed 583 gene fusions in which both partner genes contributed an annotated protein domain to the chimeric fusion protein generated by the translocation. Two thirds (66%) of these fusions were reported in hematological malignancies, 26% in mesenchymal cancers and 8% in epithelial tumors.

TICdb shows the position of each breakpoint mapped to a particular intron or exon of a specific Ensembl transcript. This enabled us to use Ensembl “Protein view”, which provides a graphical representation of the protein and of all the domains annotated in SMART, PFAM, PROSITE and PRINTS databases, in order to extract, for every gene fusion, the PFAM and PROSITE domains that are contributed to the fusion protein by each one of the partner genes. When PFAM and PROSITE domains overlapped and/or had the same INTERPRO accession number, we considered the PFAM entry only. Unique PROSITE domains without INTERPRO annotations were ignored; these include low complexity regions such as proline-rich, serine-rich or glutamine-rich regions. Coiled coil regions were included in the analysis, since they are important oligomerization domains used in many fusion proteins.

An important consideration about protein domains in native proteins is that many domains are generally found in combination with other domains in the same protein. This means that fusion proteins will usually receive two or more domains from each one of the translocation partners. In these cases, it is difficult to establish whether only one (and which) of the domains is responsible for the oncogenic properties of the fusion protein, or whether it is that particular combination of domains that is responsible for oncogenic activity. For this reason, we grouped domains into domain architectures, that is, groups of domains that are found together in the same native proteins according to Pfam annotations. Two architectures, EAD and COIL, were particularly difficult to assign. The first includes the EWS activation domain, which is not annotated as a protein domain in PFAM but has been shown to be responsible for the transforming potential of fusion proteins containing this part of the EWS protein. Interestingly, this domain is also detected, by sequence similarity, in the FUS and TAF15 proteins, which form fusion proteins with architectures found in EWS fusions. With respect to the coiled coil domain, it is present in many proteins but also lacks an annotation in protein domain databases. It appears in fusion proteins either alone or in combination with other domains, so it is not always clear whether the transforming potential of the fusion protein is due to the oligomerization properties of the coiled coil or to the combined presence of this domain with other protein domains. For this reason, we created one architecture (COIL) for those fusion proteins in which the coiled coil is the only domain present, plus various other architectures (COIL/other) for those cases in which other domains are found in combination with coiled coils. The NUP architecture is comprised by the GLFG repeats of the NUP protein.

We then generated a list of domain architectures that are brought together to the same fusion protein. These pairs of domain architectures were visualized as networks in which nodes represent a domain architecture, and edges link those architectures that are present in the same fusion protein. Networks were created using Cytoscape 2.5 (http://cytoscape.org/). Analysis of network parameters was performed using the NetworkAnalyzer plugin [16]. Supplementary Table S1 lists all architectures with the domains comprising each architecture.

The Ensembl “Protein view” also shows the reading frame in which every coding exon starts and ends (boxes shown in Figure 1). Since all translocations in TICdb are mapped to specific introns or exons, we were able to check whether the exons flanking a translocation breakpoint have compatible reading frames, that is, if the last exon of the 5′ partner gene ends in the same reading frame in which the first exon of the 3′ partner gene starts. As shown in Figure 1, this can be used to infer whether the fusion gene resulting from the translocation would keep the reading frame from both partner genes, and thus be translated as an in-frame fusion protein. Since most of the fusion sequences analyzed are derived from fusion transcripts (spliced mRNAs), these sequences already take into account potential exon skipping or alternative splicing events. In 43 of the 583 gene fusions analyzed, the reading frame of both exons seemed incompatible with an in-frame fusion product, so we went back to the original sequence to check whether other mechanisms had restored the reading frame in the fusion transcript.

## Results

### Reading frame conservation

Following the strategy explained in Methods, we analyzed reading frame compatibility of exons flanking translocation breakpoints, in 583 gene fusions coding for a potential fusion protein in which with both partner genes contribute an annotated protein domain. Interestingly, the final reading frame of the 5′ exon and the starting reading frame of the 3′ exon were compatible in 540 of the fusions analyzed, thus confirming that an in-frame fusion protein was generated in 93% of the cases. In some translocations, the breakpoint fell in the middle of an exon, but even so the reading frame was kept across the fusion. This is illustrated by a set of gene fusions between *FIP1L1* (5′ gene) and *PDGFRA* (3′ gene) in which different exons of *FIP1L1* are fused to truncated versions of exon 12 of *PDGFRA* (Ensembl transcript ENST00000381354). Deletions in this exon always result in a compatible reading frame with the corresponding exons of *FIP1L1* (exons 10, 11, 12 and 13 of *FIP1L1* transcript ENST00000358575, which end in reading frames +3, +1, +2 and +3 respectively, in version 38.36 of Ensembl), leading to in-frame fusion transcripts in all four configurations.

In the remaining 43 fusions the reading frames of flanking exons were not compatible, so they would not be expected to generate an in-frame fusion protein. In these cases we went back to the original fusion sequence and found that in 31 of these (72%) the reading frame had been restored by various mechanisms such as alternative splicing, insertion of intronic regions, insertion of non-templated nucleotides or deletion of exonic nucleotides. This was particularly common in fusion proteins involving *EWSR1*, *FUS* and *TAF15*, since 48% of such fusions had incompatible reading frames that were corrected by one of these mechanisms (24 out of 50 gene fusions analyzed for these genes). After careful evaluation of the remaining 12 fusions in which reading frames were not compatible (2% of the total 583 fusions), we assumed that a functional protein product cannot be generated in these cases.

The finding that 98% of gene fusions generate transcripts that can be translated as in-frame protein products confirms that reading frame conservation is of great functional importance in oncogenic fusion proteins, since the expected frequency of compatible reading frames between two random exons (assuming equal frequencies of +1, +2 and +3 reading frames) is one third. This is a clear signature of the strong selective pressure in somatic cells that favors those fusion products capable of driving oncogenic transformation, and it has important implications in the discussion about the identity of the factors that govern the position of translocation breakpoints in cancer cells (see below).

### Protein domain architectures present in the same fusion protein

**Figure 2:**
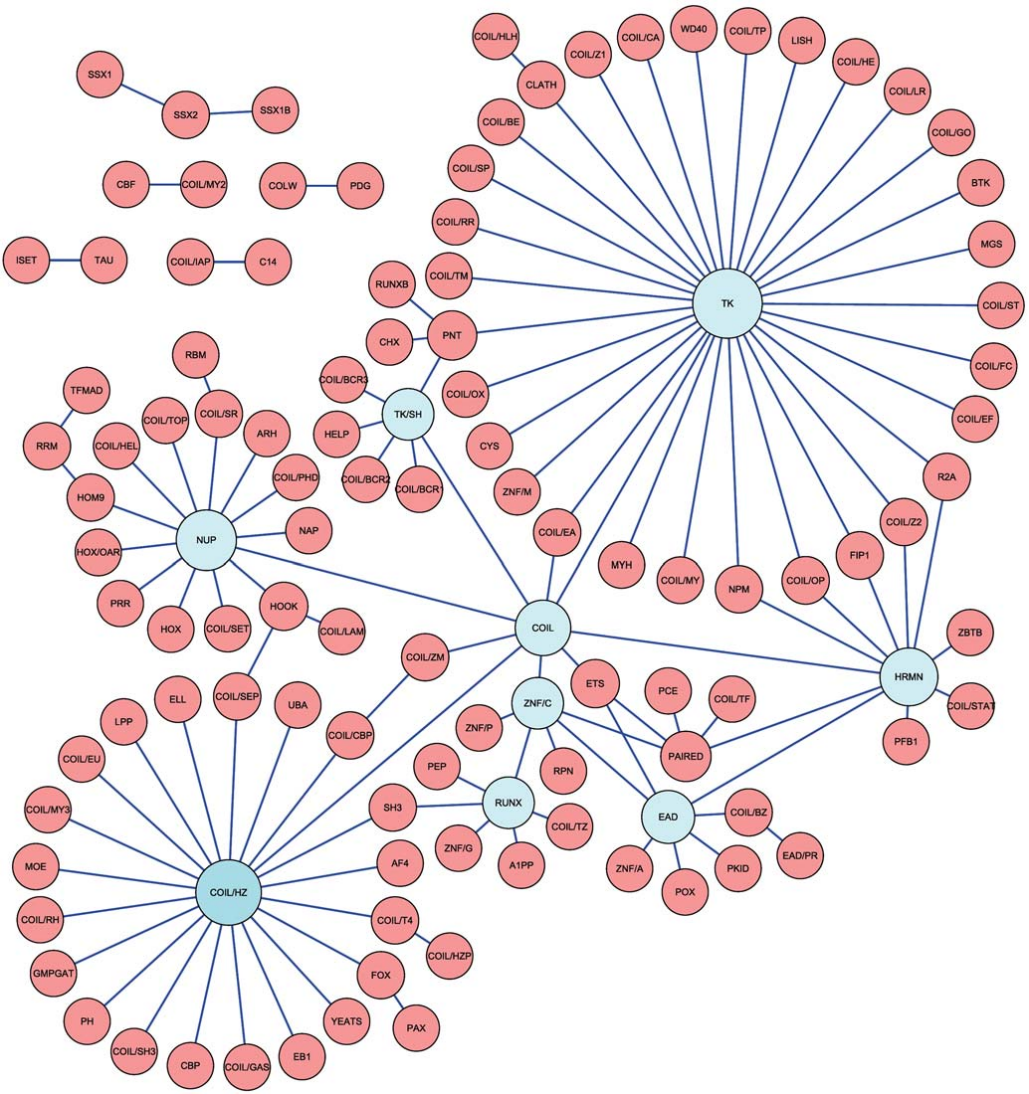
Global view of protein domain architectures in oncogenic fusion proteins. All domain architectures were merged together, resulting in a single large component plus 6 other smaller graphs. Nine nodes with more than 5 neighbors (hubs) are shown in blue. The three main gene fusion networks shown in Supplementary Figure S1 are clearly visible in the hubs corresponding to TK, NUP and COIL/HZ architectures. The size of each node is indicative of its degree (number of neighbors).

A network graph of the genes involved in chromosomal translocations that generate fusion proteins (Supplementary Figure S1) shows three main independent clusters, plus some smaller graphs that are not connected to any of the main components [15], [17]. These were analyzed as explained in the Methods section, in order to create a global network of domain architectures that are brought together to the same fusion proteins in cancer (Figure 2 and Supplementary Text S1). Topological parameters such as degree distribution show that the network of gene fusions and the network of domain architectures are both compatible with scale-free or small-world, but not random, topologies. The number of nodes (114) in the network of domain architectures (Figure 2) is less than half the number of genes rearranged in those translocations (235 nodes in Figure S1), indicating that the same architectures are used in different gene fusion events. Likewise, the network of domain architectures has a smaller diameter (8 vs. 13) and a smaller characteristic path length (3.66 vs. 4.83); as a result, network density in the network of domain architectures is more than twice the density of the network of gene fusions (0.0019 vs. 0.0008). These parameters reflect the lower complexity of the network of domain architectures with respect to the network of gene fusions. A more in-depth discussion of these networks can be found in Supplementary Text S1, Supplementary Figure S2, Supplementary Figure S3, Supplementary Figure S4 and Supplementary Figure S5.

Two interesting features are apparent in the network of domain architectures. First, all the architectures derived from the three main gene fusion networks appear as a large single graph, indicating that some domain architectures are common to all gene networks. Likewise, some of the architectures from the 21 small gene fusion graphs are also included in this large component, so that only 6 small components remain unconnected to the main network of domain architectures. Second, the more connected nodes (hubs with≥5 neighbors, shown in blue in Figure 2) identify the main classes of fusion proteins found in cancer, that is, those involving the tyrosine kinase (TK) domain, the EWS activation domain (EAD), the Runt domain, the ligand binding domain of the nuclear hormone receptor (HRMN), the AT-hook DNA binding domain (HOOK and COIL/HZ), the GLFG repeats (NUP) and coiled coils (COIL). This suggests that the network captures the main biological themes that are presently known to be used by fusion proteins in cancer.

The finding that only certain combinations of protein domains are present in oncogenic fusion proteins, forming a network of non-random topology, implies that such combinations are the result of distinct functional constraints. As mentioned above, this is a signature of the cellular selection pressures that dictate which chromosomal translocations are present in cancer cells.

In order to anticipate how this network will be affected by the discovery of new translocations, we analyzed chromosomal translocations that were published after the beginning of this work (October 2007) and thus not yet included in TICdb at the time [18]–[33]. We collected 17 gene fusions that generate a fusion protein with annotated protein domains, describing 9 new genes and 7 novel domain architectures (two of the new genes contributed an already described architecture). We also observed two novel combinations of previously described domain architectures. Additionally, in one case a 3′ partner gene *(TCF3)*, previously described as 5′ fusion partner, contributes a distinct domain architecture in this new case. The analysis of these new fusions suggests that the network of domain architectures, even though not yet complete, contains most of the architectures used by oncogenic fusion proteins, and grows at a slower rate than the network of gene fusions.

## Discussion

We have performed an unbiased survey of the literature and of all public data available to us about chromosomal translocations which create fusion proteins in human cancers. Fusions that were not informative for this analysis were excluded, namely those involved in promoter exchange (which do not create fusion proteins) and those in which one of the partner genes did not contribute a recognizable domain to the fusion protein (which are not informative for the analysis of domain co-occurrence). Therefore, it must be kept in mind that the data presented here apply to chromosomal translocations which generate fusion proteins containing protein domains annotated in Pfam. Our analysis revealed two signatures of functional selection: reading-frame compatibilty and non-random co-occurrence of protein domains. Both features might be important determinants of the position of translocation breakpoints in cancer cells. Additionally, our data could help to predict new translocations and to assess the functional relevance of novel gene fusions discovered in hematological and solid tumors.

### Role of functional selection in the position of translocation breakpoints in cancer cells

The two signatures of functional selection that we have analyzed in fusion transcripts (namely, reading frame conservation and non-random combinations of protein domains) suggest that such forces might be major factors in determining the non-random distribution of translocation breakpoints that is seen in human cancers. In this regard, the widely held view that local sequence factors are responsible for the presence of translocation breakpoints at specific genomic sites relies on the assumption that translocation breakpoints reveal the location of all DSBs generated in those cells. Thus, since translocation breakpoints are non-randomly distributed, the inference is made that DSBs are initially created non-randomly. However, sequence elements responsible for the generation of DSBs (short sequence motifs, topoisomerase II sites, dispersed repeats, intronic transcription initiation sites, cruciform structures, etc) are fairly common throughout the genome, so it is reasonable to suppose that most of the DSBs that are created across the genome during the lifetime of a somatic cell have been properly repaired and that only a small subset of misrepaired DSBs will result in oncogenic fusions and will eventually be found in tumor samples. In this respect, it must be kept in mind that the analysis of tumor samples represents an extreme case of ascertainment bias: by definition, only translocations that have been important for tumor growth will be detected, whereas many other possible translocations that did not provide a proliferative advantage to the cell will not. Some translocations, for example, would be expected to be deleterious to the cell, since two different gene alleles (one allele of each gene) have been inactivated by the breaks, so that cells carrying those translocations will eventually disappear from the tissue. Other rearrangements will be functionally neutral and the resulting fusion gene will not have an advantageous biological function. In the end, the translocations that are found in tumor samples are the result of the clonal expansion of cells that harbor translocations with the potential to promote tumor growth because they create oncogenic fusion genes. The specific breakpoints harbored by these translocations constitute the subset of non-random translocation breakpoints that are found in cancer cells.

**Figure 3:**
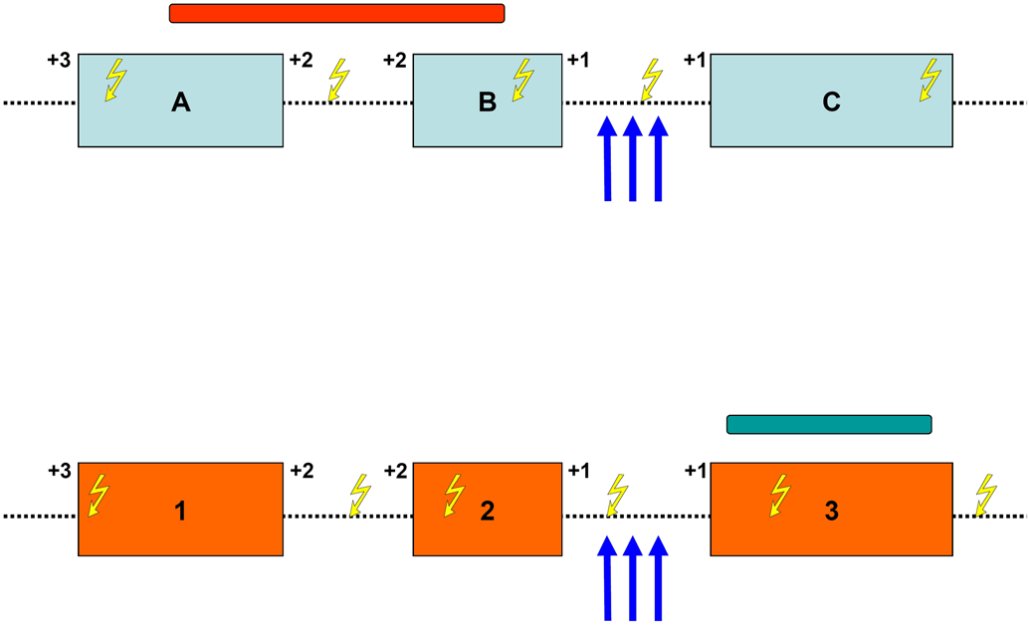
Requirements for the emergence of a successful fusion protein. Three exons of two hypothetical genes are shown (exons A, B and C in blue, exons 1, 2 and 3 in orange). The initial and final reading frame of each exon is shown (+1, +2 or +3). Exons A and B of the top gene code for a protein domain (red bar), whereas exon 3 of the bottom gene codes for another protein domain (green bar). Even if double-strand breaks (DSBs, yellow lightning symbols) were created uniformly across the sequence of both genes, only those breakpoint combinations leading to in-frame fusion proteins that code for both protein domains will display oncogenic potential. As a result, translocation breakpoints found in tumor samples will cluster to specific gene regions (vertical blue arrows).

This is illustrated in Figure 3, which shows two genes theoretically capable of participating in a reciprocal translocation with oncogenic properties, due to the domains that are present in their respective proteins. Even if the initial DSBs were distributed uniformly across those genes [34], it is obvious that not all possible translocations will create a fusion gene with oncogenic potential. Looking at the position of the regions that code for the necessary protein domains, and considering the reading frames of the various exons involved, it becomes clear that an oncogenic fusion protein will only be generated if breakpoints are located within certain introns. Other possible breakpoint combinations would lead to the loss of an important functional domain in the fusion protein, or to an out-of-frame product, and will not be favoured in tumor samples. A clear implication of this is that the perceived “non-randomness” in the genomic distribution of translocation breakpoints is not necessarily related to the initial localization of DSBs, but could be the result of the selection process by which only a few of those DSBs eventually survive in the cells of a tumor.

### Prediction of novel fusion proteins in hematological and solid tumors

If the two signatures identified in this work are important determinants of breakpoint localization, then our results should be useful for the prediction of gene fusions that have not yet been found in tumors. First, the information about which domain architectures are present in the same fusion protein can be used to select all the genes that encode a particular pair of domain architectures. This will predict several gene fusions that are potentially oncogenic. Information about the reading frames of the exons belonging to those genes should identify which specific fusions (if any) are capable of generating an in-frame fusion protein that includes the required combination of protein domains. More importantly, this analysis should also identify which introns are most likely to contain the breakpoints, and thus assist in the design of molecular strategies for the detection of those putative fusion transcripts.

One obvious implication from our work is that many potential gene fusions could generate the same combination of domain architectures, because each architecture is usually encoded by several genes. However, it is generally assumed that the majority of chromosomal translocations responsible for the development of human cancer have already been described [8], [17]. Although some new cases are published every year, most of them report novel breakpoints in previously known gene fusions, or new fusions between genes that had been previously found fused to other partners. It is not clear why many of the potential novel gene fusions have not been detected. A likely explanation is that the genes involved do not meet some of the criteria that are required for a reciprocal translocation to take place, such as proximity within the nuclear space or co-transcription in the same nuclear transcription factories [35]–[39]. Alternatively, some of these novel gene fusions might remain undetected because they were never searched for, since most studies focus on the detection of known translocations. In this regard, it is interesting to consider recent studies in which the genome of various types of cancer cells has been interrogated in an unbiased manner [40]–[43]. In the case of a diploid sample from a leukemia patient, massively parallel sequencing uncovered novel point mutations, but no genomic rearrangements [40]. End Sequence Profiling of cell lines from solid tumors revealed many somatic genomic rearrangements, but only a few of these were gene fusions. For instance, Campbell et al. [41] used massively parallel paired-end sequencing in two lung cancer cell lines and found 22 somatic interchromosomal rearrangements in the NCI-H2171 cell line, but none in NCI-H1770. Of those, only one expressed fusion transcript was identified, although it was predicted to be out-of frame. Raphael et al. [42] found one fusion between *HYDIN* gene and an anonymous gene in MCF7 metastatic breast carcinoma cell line. Another fusion between *SCL12A2* and an expressed sequence tag was found only in high passage MCF7 cells. In this same cell line, in which chromosomal aberrations have been previously described by Spectral Karyotyping (SKY) and array-Comparative Genomic Hybridization (CGH), Hampton et al. [43] found 10 gene fusions using end-sequence profiling with massively parallel sequencing. Of these, only four were found to be expressed, but their oncogenic potential was not directly tested. Considering that these studies were performed on cell lines, the number of novel expressed gene fusions is relatively low.

These recent data are also relevant to the present debate about “driver” and “passenger” mutations in cancer genomes. Due to the inherent instability of the genomes and the clonal nature of the tumorigenic process, many aberrations are expected to be found when cancer genomes are interrogated in an unbiased manner, the majority of which will be passenger aberrations with no functional relevance to the oncogenic process. In this context, there is a great need for new approaches that can distinguish those genomic changes that drive tumor initiation or progression from neutral changes that have been acquired by the clone but have no functional impact. Our results underscore two features that could be useful in this respect. However, due to the nature of the data analyzed in this work, our results are particularly relevant to hematological malignancies, which are overrepresented in our dataset (66% of all fusions studied correspond to hematological cancers). Chromosomal translocations have been difficult to find in solid tumors, particularly in epithelial cancers, due to the presence of complex karyotypes with many aberrations that are difficult to analyze [44]. However, gene fusions with clear oncogenic potential have been recently identified in prostate and lung cancers [45]–[47], suggesting that such rearrangements might be of greater importance for the development of solid tumors than generally thought. It will be interesting to see whether our findings also apply to solid malignancies, as more genomes from primary tumors are sequenced. Even if our findings are not at present entirely applicable to solid tumors, we believe that the two signatures of functional selection will be evident in expressed fusion proteins of most cancer types.

In conclusion, we identify two signatures of functional selection in oncogenic fusion proteins: specific combinations of protein domains and reading frame compatibility. Our results provide experimental support for the view of cancer as an evolutionary process at the cellular level, and highlight the importance of selection in the recurrent emergence of clonal genetic alterations and in the non-random distribution of translocation breakpoints in cancer cells. We also show that building a network of protein domain architectures that are brought together to oncogenic fusion proteins is more informative about the mechanisms involved than the network of gene fusions. The network of domain architectures is less redundant, captures most of the functional processes deregulated in oncogenesis, and will grow at a slower rate in the future. Finally, we propose that this information can be used to assess the functional relevance of novel chromosomal translocations and to predict the position of breakpoints in the genes involved.

## Supporting Information

Text S1 (0.04 MB DOC)

Table S1 List of domain architectures and of the protein domains belonging to each architecture.
(0.03 MB XLS)

Figure S1 Translocations leading to fusion proteins in cancer
(2.61 MB JPG)

Figure S2 Protein domain architectures used by translocations of the TK network
(1.26 MB JPG)

Figure S3 Protein domain architectures used by translocations of the MLL network
(1.21 MB JPG)

Figure S4 Protein domain architectures used by translocations of the NUP network
(0.50 MB JPG)

Figure S5 Protein domain architectures used by translocations of other genes
(0.58 MB JPG)
