# Supplementary material for: Signatures of Selection in Fusion Transcripts Resulting From Chromosomal Translocations in Human Cancer
Source: PLoS One. 2009 Mar 12;4(3):e4805. doi: 10.1371/journal.pone.0004805 (PMC2653638; doi:10.1371/journal.pone.0004805)
Supplement: Text S1 — (0.04 MB DOC) [file pone.0004805.s001.doc]

**Supplementary results and discussion**

**Networks of protein domain architectures that are present in the same fusion protein.**

*TK network*

This network, shown at the top right corner in Figure S1, includes all gene fusions involving tyrosine kinase genes, *EWSR1*, *ETV6*, *RUNX1*, *PML*, *RARA* and the various partner genes fused with these. In 53 of 316 fusions studied, one of the fusion partners did not provide any recognizable protein domain. Analysis of the remaining 263 gene fusions yielded a total of 86 different protein domains. When these domains were grouped as domain architectures, we obtained a network (shown in Figure S2) in which the dimensionality is reduced with respect to the respective network of gene fusions. As expected, the number of nodes is smaller (62 architectures vs. 107 genes) because different genes provide the same domain architecture in different translocations. Compared to the network of gene translocations seen in Figure S1, the network of domain architectures affords a better insight into the functional mechanisms involved, because it shows 5 hubs (nodes with >5 edges) which identify the main classes of gene translocations: (a) translocations involving the TK (tyrosine kinase domain, PF07714) and TK/SH architectures (PF07714 plus the SH2 [PF00017] and the SH3 domains [PF00018]); (b) translocations involving the EWS activation domain (EAD architecture); (c) those involving a nuclear hormone receptor binding domain (HRMN architecture, with the ligand binding domain of nuclear hormone receptor [PF00104] and the zinc-finger C4 type domain [PF00105]); and (d) translocations involving the Runt domain (RUNX architecture containing the Runt domain of *RUNX1,* PF00853). Interestingly, *ETV6* (an important hub in the network of gene fusions) contributes two architectures (PNT and ETS) that are not very connected in this network.

It is interesting to note that the network of domain architectures includes a small component comprised by three nodes (shown at the bottom right in Figure S2), corresponding to translocations of the *NUP214* gene. These translocations are included in the TK network because one of them involves the tyrosine kinase gene *ABL1*, in which *NUP214* is the 5' partner gene and contributes a Coiled coil domain to the fusion protein. However, this is mechanistically different to the other *NUP214* translocations, in which this gene is the 3' partner and contributes the NUP architecture (see below) to the respective fusion proteins. This difference is clearly shown by this small graph containing the NUP, NAP and COIL/SR architectures, where NAP and COIL/SR are contributed by *SET* and *DEK* genes, respectively.

Comparison of some simple parameters between both networks (domain architectures vs. gene fusions) also shows their different properties. For example, the average number of neighbors is slightly greater in the network of domain architectures (2.19) than in the network of translocated genes (2.13). A normalized measure of this is network density (values between 0 and 1), which is almost twice as great (0.036 and 0.020, respectively). The neighborhood connectivity distribution (neighborhood connectivity of a node is defined as the average connectivity of all its neighbors) is similar, and indicates that edges between low-connected and highly-connected nodes prevail in both networks. The network of domain architectures has a smaller diameter (8 vs. 13) and a smaller characteristic path length (3.67 vs. 5.45), indicating that the expected distance between two connected nodes is shorter than in the network of translocated genes. All these differences support the fact that the network of domain architectures reduces the complexity of the network of gene fusions.

*MLL network*

We studied 285 translocations included in the MLL network shown in Figure S1. Of these, 48 fusions showed no evidence of generating a fusion protein, so only 237 fusions were used in the analysis of protein domain architectures. In this case both networks show a very similar topology, with a central node connected to multiple nodes. As shown in Figure S3, the central architecture (corresponding to the MLL part of the fusion proteins) is COIL/HZ, which includes a Coiled coil domain, the Znf_CXXC domain (PF02008) and the AT_hook DNA binding domain (PF02178). As expected, the number of nodes is smaller in the network of domain architectures (26 vs. 48 in the network of translocated genes), because several genes contribute the same domain architecture to different translocations.

The only parameter clearly different between both networks is network density, which is twice as great in the network of domain architectures (0.08 vs. 0.04) due to a shorter characteristic path length and a smaller network diameter.

*NUP98 and other networks*

Twenty-eight gene fusions were analyzed in which *NUP98* was the 5' translocation partner, involving 22 partner genes (Figure S1). All protein domains present in these fusion proteins can be grouped into 12 domain architectures (Figure S4). The central hub of this network is the NUP architecture, comprised by the GLFG repeats of the NUP protein. Again, the network of domain architectures is very similar to the network of gene translocations, even though it contains fewer nodes.

The remaining 70 translocations analyzed are organized as isolated gene pairs, triplets or small graphs involving 59 different genes, shown as 21 separate components in Figure S1. These fusions yielded 28 different domain architectures that can be visualized as 12 distinct graphs (Figure S5).

**Special features of selected domain architectures**

*TK, TK/SH and COIL architectures*

Chromosomal translocations that target genes encoding tyrosine kinases generate fusion proteins in which the catalytic domain of the tyrosine kinase is brought together with oligomerization domains that trigger dimerization, resulting in constitutive activation of the tyrosine kinase function (Cross and Reiter 2002). However, the protein domains encoded by the partner gene may also provide additional functions, either guiding the fusion protein to a specific subcellular compartment or modulating kinase activity (Rosnet and Birnbaum 2007). In this regard, we identified two distinct TK architectures, one comprised only by the tyrosine kinase domain (TK) and another architecture (TK/SH) which also includes the SH2, SH3 and F-actin binding domains. Likewise, the Coiled coil oligomerization domain (COIL architecture) is frequently found in combination with other domains (45 different architectures), which could modulate the oligomerization properties or the transforming potential of the fusion protein.

*PNT and ETS architectures*

As shown in the network of fused genes in Figure 1, *ETV6* is a promiscuous gene fused to several partner genes (Bohlander 2005). However, *ETV6* contributes two different architectures to fusion proteins: the PNT architecture when it is the 5' partner gene of a fusion, and the ETS architecture when it is the 3' partner gene. Interestingly, neither of these architectures forms a hub in the global network of architectures shown in Figure 2, since they are fused to a limited number of other architectures. This suggests that all gene fusions involving *ETV6* use only a few pathogenic mechanisms.

*RUNX architectures*

Core binding factor (CBF) comprises a family of heterodimeric transcription factors containing a common beta subunit (CBFB) and one of several subunits which encode the Runt domain (Bly*th et a*l. 2005). We found two different domain architectures contributed by the *RUNX1* gene, depending on whether it behaves as a 5' or 3' fusion partner. As a 5' partner, *RUNX1* contributes the RUNX architecture (Runt domain, PF00853) to fusion proteins that also contain the ZNF/C (PF00096) or ZNF/G (PF00096 plus PF00320) architectures, among others. In fusions with *ETV6*, however, *RUNX1* is the 3' partner and contributes one architecture (RUNXB) that includes the Runt domain plus the Runx inhibition domain (PF08504).

*CBFB* is involved in a chromosomal inversion that results in fusion of this gene with a smooth-muscle myosin-heavy-chain gene (*MYH11*) (Cameron and Neil 2004). In the network of architectures, this appears as a separate graph linking the CBF architecture (PF02312) and the COIL/MY2 architecture (a Coiled coil plus PF01576). Although this component is distinct from the RUNX architectures, a common pathogenesis has been suggested because the CBFB-MYH11 fusion protein might interfere with normal RUNX1 activity (Reilly 2005).

*EAD architecture*

*EWSR1* is a gene frequently rearranged in sarcomas (Xia and Barr 2005). It behaves always as 5' partner of the fusion, contributing the EAD architecture. We also observed a different architecture (EAD/PR), contributed by the *FUS* gene to fusions involving similar partner genes. Both architectures are fused to COIL/BZ, an architecture that includes a Coiled coil and the bZIP_2 domain (PF07716) and is contributed by several partner genes.

Additionally, the EAD architecture appears fused to the ETS architecture contributed by *FLI1* in the most frequent type of gene fusion involving *EWSR1* (Ladanyi 2002), and to the HRMN architecture contributed by the *NR4A3* gene in other fusions. Likewise, *TAF15* also codes for an EAD architecture, and participates in fusions with *NR4A3*. These fusions provide an interesting link between the EAD architecture and the HRMN architecture of nuclear hormone receptors (see below).

*HRMN architecture*

Fusions involving the *RARA* gene are embedded in the TK network (Figure S1), due to the fact that some *RARA* translocation partners are in turn fused to genes with tyrosine kinase activity. *RARA* is a gene rearranged in acute promyelocytic leukemia (Piaz*za et a*l. 2001), and behaves as a 3' partner that contributes the HRMN architecture comprised by nuclear hormone receptor binding domains PF00104 and PF00105. The network of domain architectures identifies one group of architectures fused to HRMN that include protein domains important for oligomerization, such as Coiled coils and various types of zinc-fingers. Interestingly, these are contributed by genes that are also fused to tyrosine kinase genes, and provide and interface between the TK and HRMN architectures. Additionally, the HRMN architecture is also contributed by *NR4A3*, a gene fused to *EWSR1* and to *TAF15* in sarcomas. As a result, this architecture is linked to EAD in the network of domain architectures.

*MLL architectures*

*MLL* is another promiscuous gene translocated in acute leukaemias with bad prognosis (Slany 2005). In all fusion proteins involving this gene, *MLL* contributes either the HOOK architecture containing the AT_hook DNA binding domain (PF02178), or the COIL/HZ architecture (the same plus a Coiled coil and the ZNF_CXXC domain [PF02008]). Its fusion partners contribute a variety of domain architectures, some of which favour oligomerization of the fusion protein. Interestingly, gene fusions between *CREBBP* and *MYST3* or *MYST4* lead to similar architectures being fused, since the 5' partner genes contribute the COIL/HZP architecture which is comprised by the same domains of the COIL/HZ architecture plus the Znf_PHD domain (PF00628).

*NUP architecture*

*NUP98* is also translocated with a relatively high number of partners, always as 5' partner in the gene fusion. As a result, it contributes the NUP architecture comprised by the GLFG repeats of its N-terminal region (Slape and Aplan 2004). *NUP98* fusion partners contribute two types of architectures to fusion proteins, those containing a homeobox-related domain or those containing oligomerization and/or DNA binding domains. As explained above, *NUP214* fusions with *SET* and *DEK* lead to fusion proteins containing the NUP architecture at the C-terminal region, because *NUP214* is the 3' partner in these translocations. The graph linking NUP to NAP and COIL/SR architectures (contributed by *SET* and *DEK*, respectively), which formed a separate component in Figure S2, is thus merged to the NUP cluster of architectures in the global network shown in Figure 2.

**References**

1 Blyth K, Cameron ER, Neil JC. (2005) The RUNX genes: Gain or loss of function in cancer. Nat Rev Cancer 5(5): 376-387.

2 Bohlander SK. (2005) ETV6: A versatile player in leukemogenesis. Semin Cancer Biol 15(3): 162-174.

3 Cameron ER, Neil JC. (2004) The runx genes: Lineage-specific oncogenes and tumor suppressors. Oncogene 23(24): 4308-4314.

4 Cross NC, Reiter A. (2002) Tyrosine kinase fusion genes in chronic myeloproliferative diseases. Leukemia 16(7): 1207-1212.

5 Ladanyi M. (2002) EWS-FLI1 and ewing's sarcoma: Recent molecular data and new insights. Cancer Biol Ther 1(4): 330-336.

6 Piazza F, Gurrieri C, Pandolfi PP. (2001) The theory of APL. Oncogene 20(49): 7216-7222.

7 Reilly JT. (2005) Pathogenesis of acute myeloid leukaemia and inv(16)(p13;q22): A paradigm for understanding leukaemogenesis? Br J Haematol 128(1): 18-34.

8 Rosnet O, Birnbaum D. (2007) Myeloproliferative disorders: Let the partner guide! Haematologica 92(6): 728-730.

9 Slany RK. (2005) When epigenetics kills: MLL fusion proteins in leukemia. Hematol Oncol 23(1): 1-9.

10 Slape C, Aplan PD. (2004) The role of NUP98 gene fusions in hematologic malignancy. Leuk Lymphoma 45(7): 1341-1350.

11 Xia SJ, Barr FG. (2005) Chromosome translocations in sarcomas and the emergence of oncogenic transcription factors. Eur J Cancer 41(16): 2513-2527.

Supplementary Table S1 – **List of domain architectures and of the protein domains belonging to each architecture.**

## Supplementary Figure 1 - Translocations leading to fusion proteins in cancer

All gene fusions studied in this work are represented as a network of interacting nodes. Genes behave as 5' translocation partners (nodes in blue), 3' translocation partners (nodes in red) or both (nodes in green). The size of each node is indicative of its degree (number of neighbors).

## Supplementary Figure 2 - Protein domain architectures used by translocations of the TK network.

Network of protein domain architectures brought together by the translocations shown in the TK network in Figure S1. Nodes with more than 5 neighbors (hubs) are shown in blue.

## Supplementary Figure 3 - Protein domain architectures used by translocations of the MLL network.

Network of protein domain architectures brought together by the translocations shown in the MLL network in Figure S1. The node shown in blue corresponds to the architecture contributed by the 5' region of *MLL* gene.

## Supplementary Figure 4 - Protein domain architectures used by translocations of the NUP network.

Network of protein domain architectures brought together by the translocations shown in the NUP network in Figure S1. One node with more than 5 neighbors is shown in blue, corresponding to the GLGF repeats contributed by the 5' region of *NUP98* gene.

## Supplementary Figure 5 - Protein domain architectures used by translocations of other genes.

Network of protein domain architectures brought together by the translocations shown in the smaller component in Figure S1. These architectures are organized in 12 different components, with no hubs.
