# Supplementary figures and images for: Signatures of Selection in Fusion Transcripts Resulting From Chromosomal Translocations in Human Cancer
Source: PLoS One. 2009 Mar 12;4(3):e4805. doi: 10.1371/journal.pone.0004805 (PMC2653638; doi:10.1371/journal.pone.0004805)

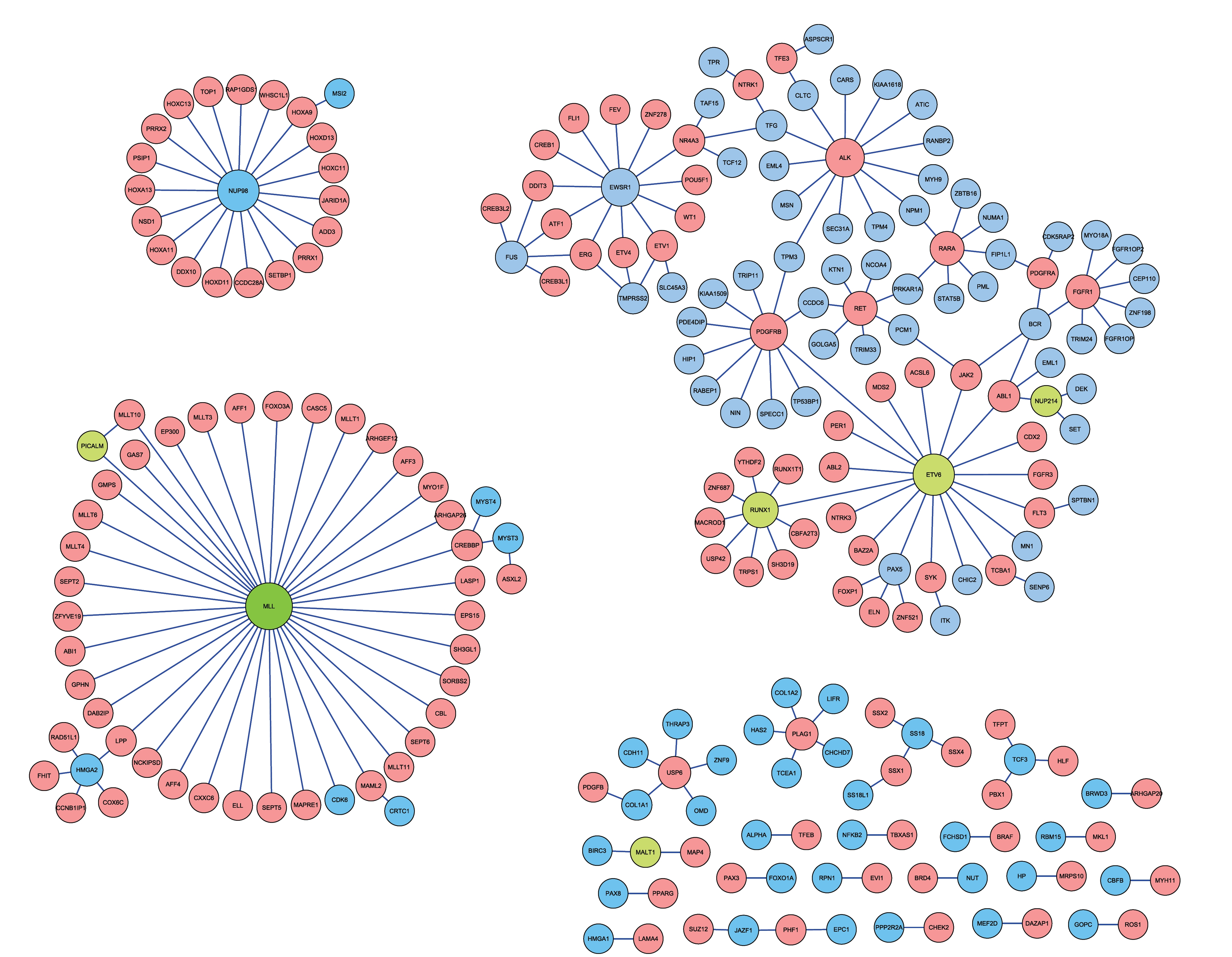

Supplement: Figure S1 — Translocations leading to fusion proteins in cancer (2.61 MB JPG) [file pone.0004805.s003.jpg]

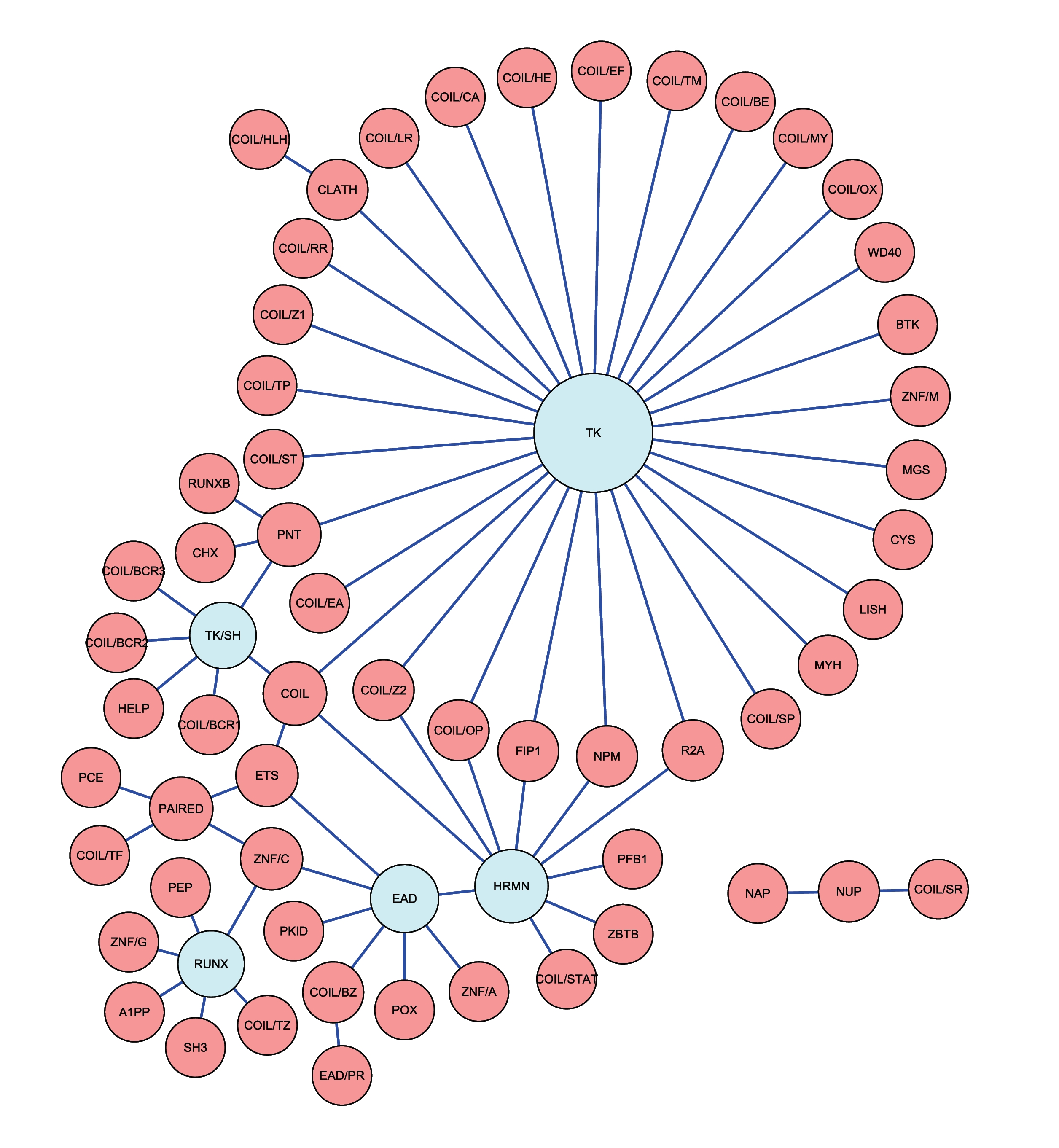

Supplement: Figure S2 — Protein domain architectures used by translocations of the TK network (1.26 MB JPG) [file pone.0004805.s004.jpg]

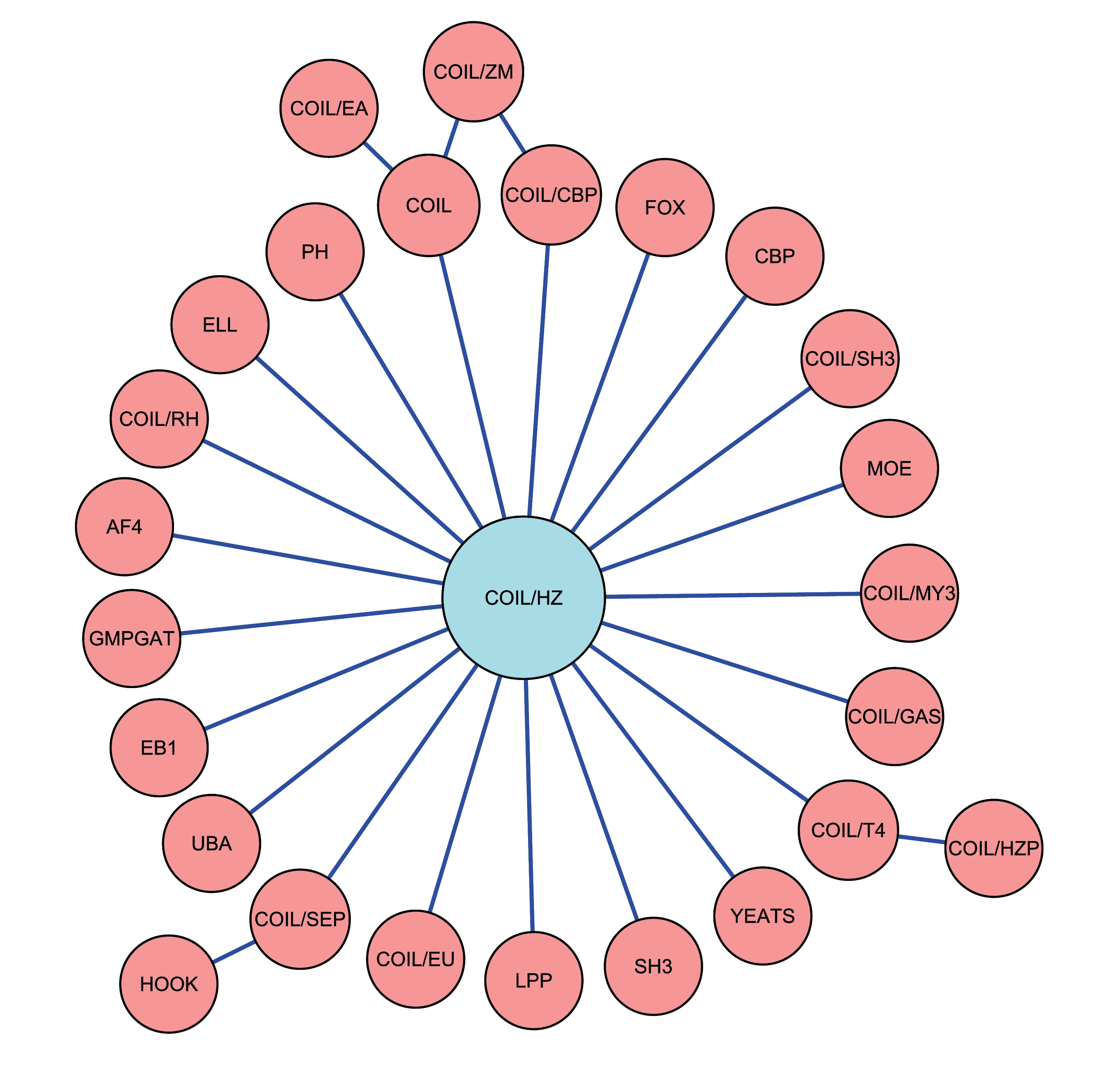

Supplement: Figure S3 — Protein domain architectures used by translocations of the MLL network (1.21 MB JPG) [file pone.0004805.s005.jpg]

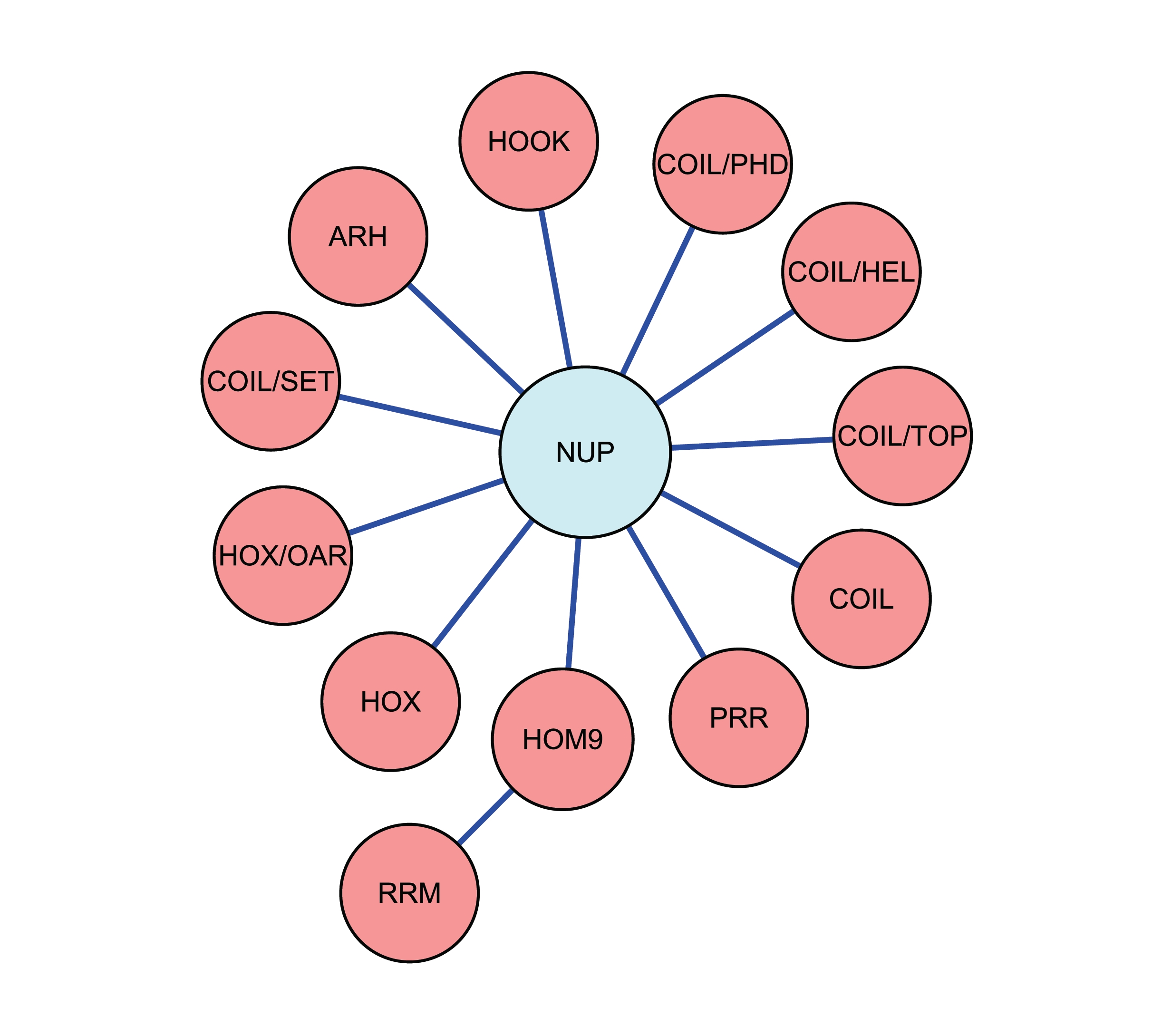

Supplement: Figure S4 — Protein domain architectures used by translocations of the NUP network (0.50 MB JPG) [file pone.0004805.s006.jpg]

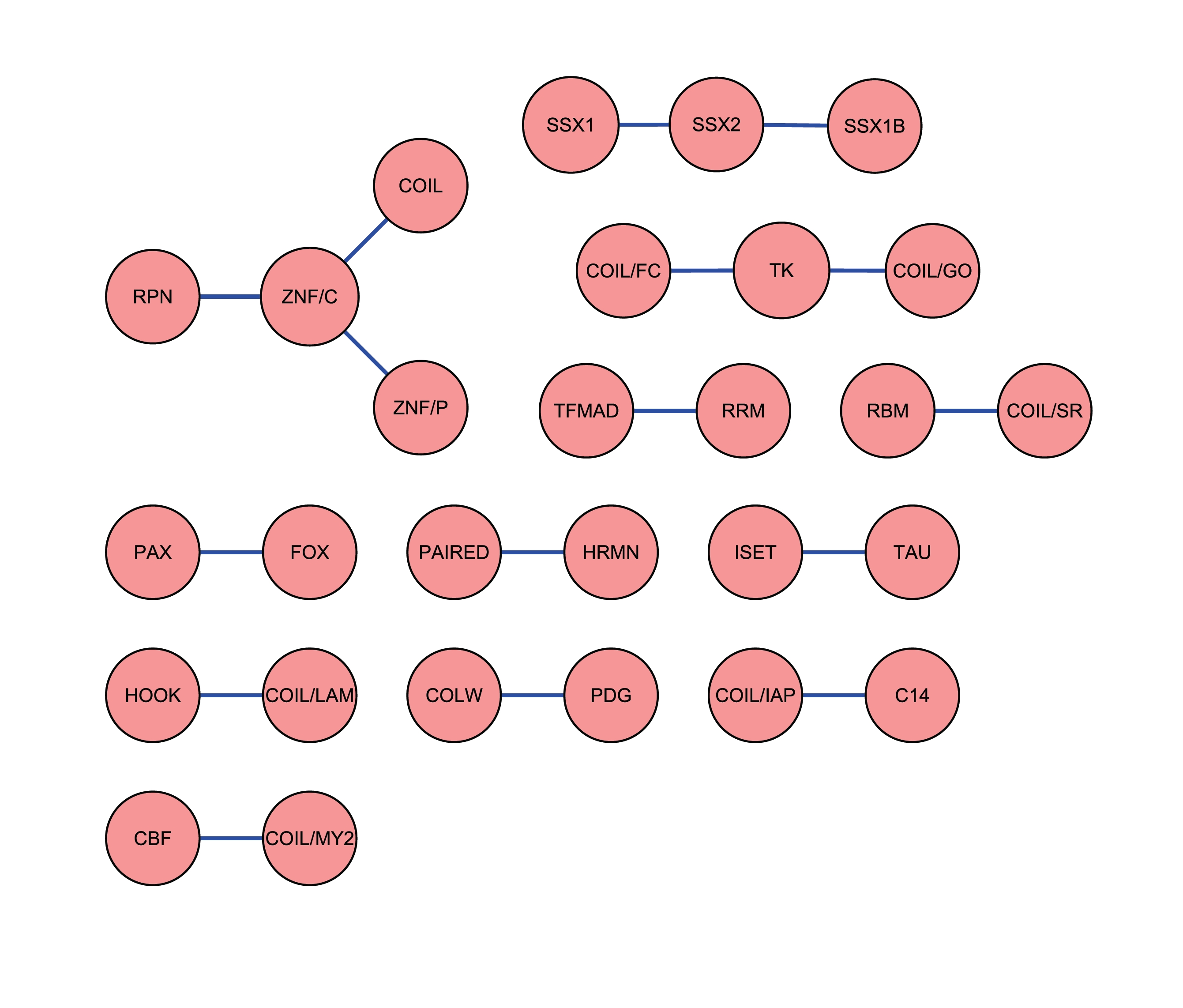

Supplement: Figure S5 — Protein domain architectures used by translocations of other genes (0.58 MB JPG) [file pone.0004805.s007.jpg]
